# Microbiomes of stony and soft deep-sea corals share rare core bacteria

**DOI:** 10.1186/s40168-019-0697-3

**Published:** 2019-06-10

**Authors:** Christina A. Kellogg

**Affiliations:** 0000000121546924 grid.2865.9 St. Petersburg Coastal and Marine Science Center, US Geological Survey, 600 4th Street South, St. Petersburg, FL 33701 USA

**Keywords:** Coral microbiome, *Lophelia pertusa*, Gorgonian, Octocoral, Conserved core

## Abstract

**Background:**

Numerous studies have shown that bacteria form stable associations with host corals and have focused on identifying conserved “core microbiomes” of bacterial associates inferred to be serving key roles in the coral holobiont. Because studies tend to focus on only stony corals (order *Scleractinia*) or soft corals (order *Alcyonacea*), it is currently unknown if there are conserved bacteria that are shared by both. A meta-analysis was done of 16S rRNA amplicon data from multiple studies generated via identical methodology to allow direct comparisons of bacterial associates across seven deep-sea corals, including both stony and soft species: *Anthothela grandiflora*, *Anthothela* sp., *Lateothela grandiflora*, *Lophelia pertusa*, *Paramuricea placomus*, *Primnoa pacifica*, and *Primnoa resedaeformis*.

**Results:**

Twenty-three operational taxonomic units (OTUs) were consistently present in greater than 50% of the coral samples. Seven amplicon sequence variants (ASVs), five of which corresponded to a conserved OTU, were consistently present in greater than 30% of the coral samples including five or greater coral species. A majority of the conserved sequences had close matches with previously identified coral-associated bacteria. While known to dominate tropical and temperate coral microbiomes, *Endozoicomonas* were extremely rare or absent from these deep-sea corals. An *Endozoicomonas* OTU associated with *Lo. pertusa* in this study was most similar to those from shallow-water stony corals, while an OTU associated with *Anthothela* spp. was most similar to those from shallow-water gorgonians.

**Conclusions:**

Bacterial sequences have been identified that are conserved at the level of class Anthozoa (i.e., found in both stony and soft corals, shallow and deep). These bacterial associates are therefore hypothesized to play important symbiotic roles and are highlighted for targeted future study. These conserved bacterial associates include taxa with the potential for nitrogen and sulfur cycling, detoxification, and hydrocarbon degradation. There is also some overlap with kit contaminants that need to be resolved. Rarely detected *Endozoicomonas* sequences are partitioned by whether the host is a stony coral or a soft coral, and the finer clustering pattern reflects the hosts’ phylogeny.

**Electronic supplementary material:**

The online version of this article (10.1186/s40168-019-0697-3) contains supplementary material, which is available to authorized users.

## Background

Numerous shallow-water studies of stony and soft corals, both tropical and temperate, have shown that bacteria form stable associations with host coral species [1–6]. Studies of cold-water corals from the deep ocean (also referred to as deep-sea corals) have also shown conserved bacterial communities that differ between coral species [7–11]. Within those coral-associated bacterial communities, individual conserved bacterial associates began to be identified in clone library studies [2, 12, 13]. With the increase in sequencing depth afforded by second-generation sequencing, studies began focusing on identifying “core microbiomes” of bacterial associates consistently found in some percentage, preferably 100%, of samples of a particular coral [14–16]. These conserved bacterial associates are inferred to be serving key roles in the coral holobiont, and therefore, identifying and studying them should yield insights into coral biology and microbial symbiosis.

The conserved core microbiome in corals has frequently been found to include *Endozoicomonas* [17–20]. Further, bacteria from the genus *Endozoicomonas*, or within the same family, have often been found to dominate shallow-water coral microbiomes both tropical and temperate [17, 18, 20–26]. However, recent studies of deep-sea corals, both stony and soft, have found *Endozoicomonas* to be rare or undetected in their microbiomes [10, 11, 27, 28]. This raises questions about the important role this bacterial group is hypothesized to play in corals and why this group is largely absent in deep-sea corals.

Ainsworth et al. recently identified rare but consistent core bacterial associates shared across multiple stony coral genera in tropical and mesophotic habitats, although at a level of 30–50% of the samples rather than 100% [29]. However, because studies tend to focus on only stony corals (order *Scleractinia*) or soft corals (order *Alcyonacea*), it is currently unknown if there are conserved bacteria that are shared by both. Tackling the question is further impeded by the differences in extraction method, primer choice, and sequencing platform between studies on different corals, limiting our ability to compare across hosts.

In this study, I tested the hypothesis that there are conserved bacterial associates present across both stony and soft deep-sea corals by reanalyzing aggregated data from previous deep-sea coral investigations [11, 27, 28, 30]. Further, I investigated rare *Endozoicomonas* sequences present in some of these corals to determine how similar they are to the sequences derived from tropical and temperate coral microbiomes. Pyrosequencing of 16S ribosomal rRNA amplicons using identical methodology from extraction to sequencing in all of the aggregated studies allows for the first time a direct comparison of bacterial associates across seven species of deep-sea corals: *Anthothela grandiflora*, *Anthothela* sp., *Lateothela grandiflora*, *Lophelia pertusa*, *Paramuricea placomus*, *Primnoa pacifica*, and *Primnoa resedaeformis*.

## Methods

### Sample data

Datasets of 16S rRNA amplicons and associated environmental data from four prior publications covering seven species of deep-sea corals were combined and reanalyzed in aggregate (Additional file 1; [11, 27, 28, 30]). These raw sequence data are available from the NCBI Sequence Read Archive under BioProjects PRJNA296835, PRJNA297333, PRJNA305617, and PRJNA348705 as well as USGS data releases for each of the original papers [31–34].

### DNA extraction and 16S rRNA gene amplicon sequencing

Uniform methods were used across these four studies to preserve, extract, and sequence bacterial amplicons associated with the corals. Briefly, coral samples were preserved in the field using RNAlater and stored at − 80 °C until processed. DNA was extracted using the MO BIO Powerplant DNA Isolation Kit with the modifications suggested by Sunagawa et al. [4, 35]. DNA samples were amplified with primers 563F (5′-AYTGGGYDTAAAGNG) and 926R (5′-CCGT CAATTYYTTTRAGTTT) which target the V4-V5 hypervariable region of the 16S rRNA gene [36]. Samples were pyrosequenced using Roche 454 GS FLX Titanium chemistry.

### Sequence analysis of amplicon datasets

Bioinformatic analysis was conducted using QIIME 1.9.1 [37] and DADA2 1.9.2 [38] following the workflow of specific scripts and parameters for each step listed in Additional file 2. In brief, for QIIME, individual datasets had been previously denoised and trimmed of primers, and the resulting files were combined into a single fasta file for an open-reference operational taxonomic unit (OTU) picking [39]. Greengenes release 13_8 was used as the reference database [40, 41], and chimeras were removed by usearch61 [42]. Non-bacterial sequences and singletons were filtered out. A non-rarefied OTU table (Additional file 3) was used to determine the core OTUs shared across multiple coral species. Samples were then randomly rarefied to the size of the smallest library (4287 sequences) before the diversity metrics were calculated [43]. In brief, for DADA2 (run in RStudio using R version 3.5.1 [44]), the Roche 454 ssf files were converted to individual fastq files using QIIME scripts, and primers were removed using Biostrings [45] and Cutadapt [46]. Reads were trimmed to 325 bp based on quality profiles. Sequences were processed through the filter and inference modules of DADA2 in groups corresponding to original 454 runs (6 separate runs total) in order to more accurately estimate the error rates. The data were then merged for the chimera removal and taxonomy assignment. Greengenes release 13_8 was used as the reference database to keep the consistency with the OTU taxonomy. Amplicon sequence variants (ASVs) that were unclassified below the domain *Bacteria* level were removed. The resulting ASV table (Additional file 4) contains 4299 ASVs.

### Sequence analysis of *Endozoicomonas* sequences

Operational taxonomic units that were classified by Greengenes as belonging to *Endozoicomonadaceae* were identified from the OTU table (Additional file 3). The *Endozoicomonadaceae* OTU sequences and those of reference clone library sequences previously derived from corals were aligned using Clustal X [47]. Stony corals that had comparable reference *Endozoicomonas* sequences included *Acropora humilis* (Genbank Accession KC668469.1) [22], *Montipora aequituberculata* (FJ347758.1) [48], *Pocillopora damicornis* (KC668770.1) [22], and *Stylophora pistillata* (KC669131.1) [22]. Soft corals that had comparable reference *Endozoicomonas* sequences included *Eunicella cavolini* (JQ691583.1) [21] and *Gorgonia ventalina* (GU118516.1) [4]. An online phylogenetic tree viewer was used to visualize the relationships revealed by the alignment [49].

### Statistical analyses

Alpha and beta diversity metrics and relative abundance summaries were calculated within QIIME 1.9.1 [37]. Community similarity was assessed by principal coordinate analysis (PCoA) using weighted and unweighted Unifrac, Bray-Curtis, and Binary Sorenson Dice to determine the importance of taxonomic and phylogenetic relationships and sequence abundance. PERMANOVA analyses were conducted using PRIMER v7 software [50] on the Binary Sorenson Dice distance matrix from QIIME. A one-factor test design was based on the partial sum of squares type III, 9999 permutations of residuals under an unrestricted permutation of raw data. A two-factor test was based on the same parameters but with the factor “species” nested under the factor “genus.” Core microbiomes were identified based on the presence of an OTU in > 50% of the coral samples (*n* = 51) or the presence of an ASV in > 30% of the coral samples (*n* = 51) with the additional caveat of being present in > 5 coral hosts. This additional caveat was necessary to avoid detection of sequences that were conserved only at the level of species or genus.

## Results

A total of 2,323,795 amplicon sequences were processed from 66 samples across 7 species of deep-sea corals (Additional file 1). After filtering and removal of low-read samples, this was reduced to 2,205,336 sequences across 51 samples: 12 samples of *A. grandiflora*, 4 samples of *Anthothela* sp., 3 samples of *Anthothela* ND (species not determined by genetics—either *A. grandiflora* or *Anthothela* sp.), 1 sample of *L. grandiflo*ra, 12 samples of *Lo. pertusa*, 3 samples of *P. placomus*, 6 samples of *Pr. pacifica*, and 10 samples of *Pr. resedaeformis* (Additional file 5).

### Core bacterial associates

The final OTU table, generated after filtering to remove non-bacterial sequences but prior to rarefaction (3204 OTUs; Additional file 3), was searched for conserved OTUs across all 51 samples of the 7 deep-sea corals. While there were no OTUs that were present in 100% of the samples, there were 23 OTUs present in more than 50% of the coral samples (Table 1). The majority of these 23 OTUs could only be identified to the order or family level, and one was unassignable even to a phylum. The final ASV table, generated after filtering to remove sequences unclassified beyond the domain level (4299 ASVs; Additional file 4) was also searched for conserved ASVs, uncovering 7 ASVs that were present in more than 30% of coral samples, across at least 5 of the 7 species (Table 2). Five of these ASVs were the same sequences as conserved OTUs identified in Table 1. A majority (80%) of these conserved sequences had close matches (≥ 96% identity) with previously identified coral-associated bacteria (Table 3).

**Table 1:** Conserved bacterial OTUs present in > 50% of coral samples (*n* = 51)

OTU	Percent of coral samples	Assignment UCLUST	Found in coral hosts^‡^
4447394/4462014	92	*Actinobacteria*: family *Propionibacteriaceae*, genus *Propionibacterium*^♣^	1–7 (all)
245163	84	*Planctomycetes*: family *Pirellulaceae*	1, 2, 3, 4, 6, 7
1915223	76	*Planctomycetes*: family *Pirellulaceae*	1–7 (all)
4408871	69	*Planctomycetes*: family *Pirellulaceae*	1–7 (all)
4483490	69	*Betaproteobacteria*: family *Comamonadaceae*, genus *Acidovorax*^♣^	1–7 (all)
New.ReferenceOTU98	63	*Planctomycetes*: order *Phycisphaerales*	1, 2, 3, 4, 5
156342	61	*Alphaproteobacteria*: order *Kiloniellales*	1, 2, 3, 5, 6, 7
4475561	61	*Alphaproteobacteria*: family *Bradyrhizobiaceae*^♣^	1–7 (all)
New.ReferenceOTU33	61	Unassigned	1, 2, 6, 7
New.CleanUp.ReferenceOTU8	61	*Epsilonproteobacteria*: order *Campylobacterales*	1, 2, 3, 6, 7
New.ReferenceOTU21	59	*Gammaproteobacteria*: family *Moraxellaceae*, genus *Acinetobacter*^♣^	1, 2, 3, 6, 7
4455242	55	*Gammaproteobacteria*: family *Vibrionaceae*	1, 2, 3, 5, 6, 7
2222982	55	*Planctomycetes*: family *Pirellulaceae*	1–7 (all)
226495	55	*Planctomycetes*: family *Pirellulaceae*	1, 3, 4, 5, 6, 7
160569	55	*Planctomycetes*: order *Phycisphaerales*	1, 2, 3, 4, 5, 7
New.ReferenceOTU66	55	*Gammaproteobacteria*: family *Moraxellaceae*, genus *Acinetobacter*^♣^	4, 6, 7
New.ReferenceOTU53	55	*Gammaproteobacteria*: family *Pseudomonadaceae*, genus *Pseudomonas*^♣^	4, 6, 7
4307347	53	*Alphaproteobacteria*: family *Methylobacteriaceae*^♣^	4, 5, 6, 7
4457268	53	*Gammaproteobacteria*: family *Enterobacteriaceae*^♣^	4, 5, 6, 7
4371886	53	*Gammaproteobacteria*: family *Xanthomonadaceae*, genus *Lysobacter*	4, 5, 6, 7
4308875	53	*Planctomycetes*: order *Phycisphaerales*	1–7 (all)
4461879	53	*Gammaproteobacteria*: family *Xanthomonadaceae*	1, 2, 3, 5, 6, 7
New.ReferenceOTU4	51	*Alphaproteobacteria*: order *Kiloniellales*	1, 2, 3, 6, 7

^‡^Coral hosts: 1, *Anthothela grandiflora*; 2, *Anthothela* sp.; 3, *Lateothela grandiflo*ra; 4, *Lophelia pertusa*; 5, *Paramuricea placomus*; 6, *Primnoa pacifica*; 7, *Primnoa resedaeformis*

^♣^Bacterial families/genera that are commonly found as kit contaminants

**Table 2:** Conserved bacterial ASVs present in > 30% of coral samples (*n* = 51) and present in at least 5 out of 7 coral species. When an ASV sequence is a 99–100% match to the representative OTU sequence from Table 1, it is identified in parentheses

OTU	Percent of coral samples	Assignment UCLUST	Found in coral hosts^‡^
ASV_50 (OTU 4462014)	63	*Actinobacteria*: family *Propionibacteriaceae*, genus *Propionibacterium*^♣^	1–7 (all)
ASV_53 (OTU 4475561)	53	*Alphaproteobacteria*: family *Bradyrhizobiaceae*, genus *Bradyrhizobium*^♣^	1–7 (all)
ASV_58 (OTU 245163)	53	*Planctomycetes*: family *Pirellulaceae*	1, 2, 3, 4, 5, 7
ASV_21	43	*Firmicutes*: family *Bacillaceae*, genus *Bacillus*^♣^	3, 4, 5, 6, 7
ASV_75 (OTU 226495)	37	*Planctomycetes*: family *Pirellulaceae*	1, 3, 5, 6, 7
ASV_140	37	*Alphaproteobacteria*: family *Sphingomonadaceae*, genus *Sphingobium*^♣^	1, 2, 3, 5, 6, 7
ASV_85 (OTU1915223)	31	*Planctomycetes*: family *Pirellulaceae*	1, 2, 3, 4, 5, 7

^‡^Coral hosts: 1, *Anthothela grandiflora*; 2, *Anthothela* sp.; 3, *Lateothela grandiflo*ra; 4, *Lophelia pertusa*; 5, *Paramuricea placomus*; 6, *Primnoa pacifica*; 7, *Primnoa resedaeformis*

^♣^Bacterial families/genera that are commonly found as kit contaminants

**Table 3:** Conserved bacterial OTUs/ASVs with highly similar sequences to other coral microbiomes

OTU/ASV	Assignment UCLUST	Corals with similar bacterial sequences	Accession number	Percent identity
4447394/4462014 ASV_50, ASV_163, ASV_1083, ASV_1446, ASV_936, ASV_680, ASV_1526, ASV_1089	*Actinobacteria*: family *Propionibacteriaceae*, genus *Propionibacterium*^♣^	*Acropora palmata*	EU861208.1	98–100
		*Fungia granulosa*	**DQ097293.1**	95–100
		*Lophelia pertusa*	AM911348.2	98–100
		*Orbicella annularis*	DQ200498.1	99–100
		*Orbicella faveolata*	JQ516491.1	98–100
		*Orbicella faveolata*	JQ516688.1	99
		*Orbicella faveolata*	JQ516384.1	97–98
		*Pocillopora meandrina*	EU249959.1	97–99
		*Pocillopora meandrina*	EU249977.1	97–99
		*Porites compressa*	FJ930453.1	98
		*Porites lutea*	KF179755.1	99
		*Porites lutea*	KP305502.1	98–100
		*Porites lutea*	KP305839.1	98–99
		*Siderastrea stellata*	JF835676.1	97–98
		“Coral”	KY393360.1	98–100
		“Coral”	KY393364.1	97–98
245163 ASV_58, ASV_185, ASV_407, ASV_75, ASV_377	*Planctomycetes*: family *Pirellulaceae*	*Muricea elongata*	DQ917853.1	98–100
1915223 ASV_116, ASV_127, ASV_85, ASV_373, ASV_178, ASV_2905, ASV_2832	*Planctomycetes*: family *Pirellulaceae*	*Astrangia poculata*	PRJNA380119	99
		*Gorgonia ventilina*	GU118476.1	99
		*Orbicella annularis*	DQ200559.1	99
		*Stylophora pistillata*	KC669264.1	99
44008871 ASV_90, ASV_234, ASV_110, ASV_469	*Planctomycetes*: family *Pirellulaceae*	*Pocillopora meandrina*	EU249980.1	98–99
4483490 ASV_168, ASV_2169, ASV_1531, ASV_3865, ASV_2609	*Betaproteobacteria*: family *Comamonadaceae*, genus *Acidovorax*^♣^	*Porites cylindrica*	GQ413900.1	97–99
		*Siderastrea stellata*	JF835733.1	97–99
		*Siderastrea stellata*	JF835697.1	98–99
		*Siderastrea stellata*	JF835673.1	97–98
		*Siderastrea stellata*	JF835695.1	98
		*Stephanocoenia intersepta*	KC190250.1	99
		*Stephanocoenia intersepta*	KC190258.1	99
		*Tubastraea coccinea*	JF925026.1	98–100
4475561 ASV_53	*Alphaproteobacteria*: family *Bradyrhizobiaceae*^♣^	*Acropora cervicornis*	KC737030.1	99
		*Acropora cervicornis*	GU117999.1	99
		*Acropora palmata*	GU118021.1	99
		*Gorgonia ventilina*	GU118506.1	99
		*Porites* sp.	DQ309378.1	100
		*Pseudodiploria strigosa*	GU118187.1	99
156342 ASV_11	*Alphaproteobacteria*: order *Kiloniellales*	Bamboo coral	DQ395873.1	99
			DQ395762.1	99
			DQ395662.1	99
			DQ395906.1	98
New.CleanUp.ReferenceOTU8 ASV_112	*Epsilonproteobacteria*: order *Campylobacterales*	*Acropora cervicornis*	GU117990.1	96
New.ReferenceOTU21 ASV_38	*Gammaproteobacteria*: family *Moraxellaceae*, genus *Acinetobacter*^♣^	“Coral”	MH744724.1	96
4455242 ASV_72	*Gammaproteobacteria*: family *Vibrionaceae*	*Alcyonium digitatum*	KT583560.1	99
		*Alcyonium digitatum*	KT583432.1	99
		*Corallium rubrum*	**HG942391.1**	99
		*Eunicella labiata*	MF461381.1	100
		*Eunicella labiata*	MF461377.1	99
		*Leptogorgia* sp.	MG099530.1	99
		*Lophelia pertusa*	**HQ640762.1**	99
		*Lophelia pertusa*	**HQ640866.1**	99
		*Oculina patagonica*	KF577097.1	99
		*Porites astreoides*	MF600122.1	100
		*Pseudoptergorgia* sp.	GQ406789.1	99
226495 ASV_75	*Planctomycetes*: family *Pirellulaceae*	*Muricea elongata*	DQ917853.1	100
New.ReferenceOTU66 ASV_47, ASV_3281, ASV_186, ASV_587, ASV_139, ASV_821, ASV_2980	*Gammaproteobacteria*: family *Moraxellaceae*, genus *Acinetobacter*^♣^	*Madracis decactis*	**KY914393.1**	98–100
		*Pocillopora damicornis*	AY700608.1	98–99
		*Porites* sp.	KM079057.1	98–99
		*Tubastraea* sp.	**KY914396.1**	98–100
		*Tubastraea* sp.	**KY914208.1**	98–99
		*Turbinaria mesenterina*	**EU276980**	98–100
New.ReferenceOTU53 ASV_164, ASV_905, ASV_196, ASV_463, ASV_409, ASV_305, ASV_2162, ASV_1987, ASV_1521, ASV_2226	*Gammaproteobacteria*: family *Pseudomonadaceae*, genus *Pseudomonas*^♣^	*Platygyra carnosus*	**JF411506.1**	98–100
		*Tubastraea* sp.	**KY914202.1**	98–100
4307347 ASV_40, ASV_4069, ASV_1562, ASV_3638, ASV3611	*Alphaproteobacteria*: family *Methylobacteriaceae*^♣^	*Lophelia pertusa*	AM911405.1	99–100
		*Orbicella faveolata*	JQ515453.1	99
		*Porites compressa*	FJ930589.1	96–97
4457268 ASV_62	*Gammaproteobacteria*: family *Enterobacteriaceae*^♣^	*Acropora desalwii*	KJ616368	96–97
		*Fungia granulosa*	**DQ097299.1**	97
		*Orbicella faveolata*	JQ516590.1	98–99
		*Orbicella faveolata*	JQ516581.1	99–100
		*Orbicella faveolata*	JQ516578.1	98–99
		*Orbicella faveolata*	JQ516577.1	98–99
		*Orbicella faveolata*	FJ202675.1	99–100
		*Pocillopora meandrina*	EU249962.1	99–100
		*Porites compressa*	FJ930291.1	99–100
		*Tubastrea micrantha*	KJ616365.1	96–97
4371886 ASV_17, ASV1451, ASV_479	*Gammaproteobacteria*: family *Xanthomonadaceae*, genus *Lysobacter*	*Porites* sp.	KM079054.1	99–100
4461879 ASV_259, ASV_2197, ASV_406, ASV_2041	*Gammaproteobacteria*: family *Xanthomonadaceae*	*Acropora digitifera*	JN248444.1	97–100
		*Acropora digitifera*	JN248443.1	97–99
		*Acropora digitifera*	JN248442.1	97–100
		*Orbicella faveolata*	JQ516453.1	96–99
		*Pocillopora damicornis*	AY700609.1	97–99
		*Pocillopora meandrina*	EU249965.1	98–100
		*Porites* sp.	DQ309377.1	97–100
		*Sideastrea siderea*	**JF792079.1**	98–100
		*Sideastrea siderea*	**JF792069.1**	98–100
		*Siderastrea stellata*	JF835651.1	97–99
		*Tubastraea coccinea*	JF925027.1	97–99
New.ReferenceOTU4 ASV_59, ASV_1406, ASV_593, ASV_200, ASV_318, ASV_147	*Alphaproteobacteria*: order *Kiloniellales*	Bamboo coral	DQ395873.1	98–100
			DQ395762.1	98–100
			DQ395662.1	98–100
			DQ395906.1	98–100
ASV_21	*Firmicutes*: family *Bacillaceae*, genus *Bacillus*^♣^	*Acropora digitifera*	**EU660355.1**	99
		*Acropora digitifera*	**EU660327.1**	99
		*Acropora palmata*	JF346760.1	97
		*Alcyonium digitatum*	**KT583461.1**	98
		*Alcyonium digitatum*	KT583427.1	97
		*Eunicea succinea*	**MG099636.1**	98
		*Madracis decactis*	**KY914242.1**	98
		*Madracis decactis*	**KY914116.1**	97
		*Mussismilia* sp.	JN106654.1	97
		*Platygyra carnosus*	JF411582.1	97
		*Pocillopora* sp.	**MK418934.1**	98
		*Pocillopora* sp.	**MK418932.1**	98
		*Pocillopora* sp	**MK418942.1**	97
		*Pocillopora meandrina*	EU249974.1	97
ASV_140	*Alphaproteobacteria*: family *Sphingomonadaceae*, genus *Sphingobium*^♣^	*Acropora cervicornis*	KC737046.1	97
		*Lophelia pertusa*	AM911354.2	100
		*Lophelia pertusa*	AM911424.1	100
		*Siderastrea stellata*	JF835729.1	100
		*Siderastrea stellata*	JF835707.1	99
		*Siderastrea stellata*	HM216535.1	98
		*Tubastraea* sp.	**KY913986.1**	97

Bold accession numbers indicated a cultured isolate

^♣^Bacterial families/genera that are commonly found as kit contaminants

A *Propionibacterium* sequence was present in all 7 coral hosts, across 92% of the samples based on an OTU and 63% of the samples based on an ASV (Tables 1 and 2). This finding required some sleuthing, since the sequence appears under two different OTU numbers. In *Lo. pertusa*, *P. placomus*, and *Anthothela* sp., this sequence appears as OTU 4447394, whereas in the 2 *Primnoa* species, it shows up as OTU 4462014; however, the only difference between the OTUs is two additional bases (GG) at the beginning of OTU 4462014. There is a 100% sequence identity between OTU 4462014 and ASV_50. The *Propionibacterium* sequences from *Primnoa*, *Paramuricea*, and *Anthothela* have 100% identity across 331 base pairs (bp). The sequence from *Lo. pertusa* is 99% similar, sharing 217/220 bp with the other coral sequences, but containing 3 single bp insertions.

The second most commonly conserved OTU (245163, *Pirellulaceae*, found in 84% of samples and identical to a conserved ASV found in 53% of samples) was present in 6 coral species. A *Bradyrhizobiaceae* sequence (OTU4475561 and ASV_53) was found in 61% and 53% of the samples depending on the pipeline. The other broadly conserved sequences include a number of *Pirellulaceae* (as OTUs or ASVs), plus orders *Campylobacterales*, *Kiloniellales*, and *Phycisphaerales*; families *Enterobacteriaceae*, *Methylobacteriaceae*, and *Vibrionaceae*; and the genera *Acidovorax*, *Acinetobacter*, *Bacillus*, *Lysobacter*, *Pseudomonas*, and *Sphingobium* (Tables 1 and 2).

### *Endozoicomonas*

**Fig. 1:**
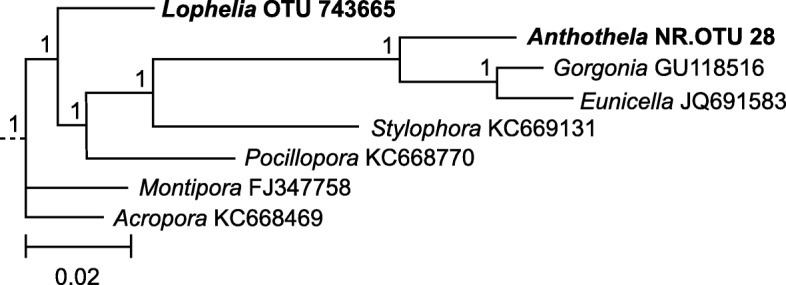
Newick phylogenetic tree visualization of the alignment of *Endozoicomonas*-like sequences. Sequences are listed by the name of the coral host. Operational taxonomic units from this study are in bold; reference sequences include NCBI accession numbers

There were 7 OTUs and 6 ASVs that were classified as belonging to the family *Endozoicomonaceae* (Additional files 3 and 4). These rare sequences were typically found in 1–7 samples, with low numbers of reads. The exceptions were OTU 743665 (ASV_56) which was found in 6 *Lo. pertusa* samples with over 100 reads each for a total of 2574 sequences, and New.ReferenceOTU28 (ASV_45) which was found in 7 *Anthothela* spp. samples with a total of 3446 sequences. When aligned with known coral-associated *Endozoicomonas* sequences derived from shallow-water stony and soft corals, *Endozoicomonaceae* OTU 743665 (*Lo. pertusa*) grouped with other stony corals and New.ReferenceOTU28 (*Anthothela* spp.) grouped closely with other soft corals (Fig. 1).

### Bacterial community composition

**Fig. 2:**
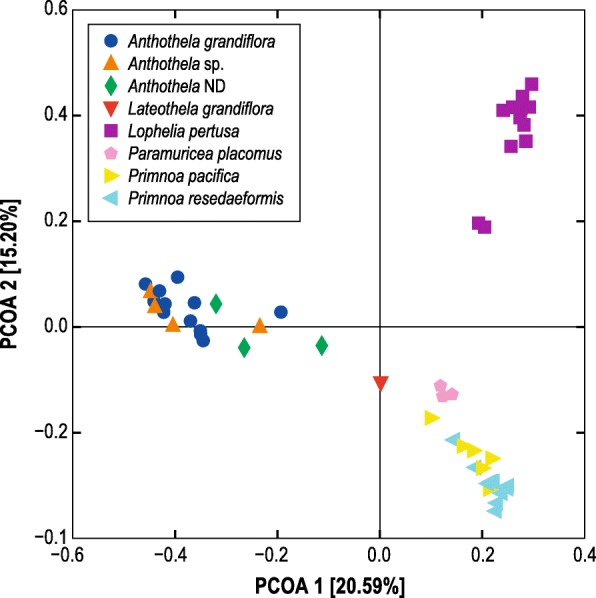
Principal coordinate analysis of coral microbiomes based on Binary Sorensen Dice distance matrix

For comparisons of the entire bacterial community composition between the 7 corals, the most informative principal coordinate analysis was that based on the Binary Sorenson Dice distance matrix (Fig. 2). The samples appear to cluster at the level of host genus, with the two *Anthothela* spp. grouping together and the two *Primnoa* spp. grouping together. To test the robustness of this pattern, I performed a PERMANOVA across corals using genus as a factor. There were significant differences between the coral genera (*P*_PERMANOVA_ = 0.0001, pseudo-*F* = 8.066, 9787 unique permutations). Subsequent pair-wise tests showed all coral genera were significantly different from each other with the exception of pairs that included *L. grandiflora*, since there is only one replicate of that coral genus and therefore the test did not have sufficient statistical power. Nesting the factor of “species” within “genus,” there were significant differences between *Pr. pacifica* and *Pr. resedaeformis* (*P*_PERMANOVA_ = 0.0005, *t* = 1.9316, 5645 unique permutations) in spite of their close clustering. Conversely, the *Anthothela* spp. group was not significantly different: *A. grandiflora* vs. *Anthothela* sp. (*P*_PERMANOVA_ = 0.3426, *t* = 1.0376, 1808 unique permutations), *Anthothela* sp. vs. *Anthothela* ND (*P*_PERMANOVA_ = 0.3186, *t* = 1.0464, 35 unique permutations), and *A. grandiflora* vs. *Anthothela* ND (*P*_PERMANOVA_ = 0.0238, *t* = 1.3249, 455 unique permutations).

## Discussion

Using the most stringent criteria (presence in 100% of samples), previous studies of each of these 7 deep-sea coral species identified from 1 to 48 bacterial taxa that constituted a conserved core [11, 27, 28, 30]. However, in most cases, this was done using rarefied datasets, which could lead to an underestimation if in fact the OTUs were present in the randomly discarded sequences. This meta-analysis across all 7 corals identified 23 highly conserved OTUs based on an unrarefied OTU table and an additional 2 conserved ASVs in addition to ASVs that matched the conserved OTUs. Eighty percent of these sequences (20/25) were highly similar (≥ 96% identity) to sequences previously identified as coral-associated (Table 3).

### Conserved vs. contaminants

It is problematic that acquisition of these sequence datasets predated the 2014 publication by Salter et al. [51] that triggered widespread recognition of the need to run kit blank controls to detect possible contamination from DNA extraction kits. Such controls are becoming a common practice and are particularly critical for Illumina datasets which are an order of magnitude deeper than 454 datasets such as the ones compiled in this study. Not having kit controls for these datasets means having to interpret the findings with caution; however, it would be an oversimplification to automatically assume that the presence of certain bacterial groups that have been detected as contaminants in some kits indicates contamination [51, 52]. Even studies that have identified kit contaminants acknowledge that many of the contaminants detected can be indistinguishable from bacteria genuinely present in samples and that arbitrarily discarding low prevalence microbes to “correct” for contamination could hinder the identification of relevant minor components of microbiomes [52]. Bearing this in mind, I have marked the 8 OTUs in Table 1 and 4 ASVs in Table 2 that represent bacterial families/genera also commonly found as kit contaminants. All of these bacterial groups (*Acidovorax*, *Acinetobacter*, *Bacillus*, *Bradyrhizobiaceae*, *Enterobacteriaceae*, *Propionibacterium*, *Pseudomonas*, *Methylobacteriaceae*, *Sphingobium*) have commonly been found in coral microbiomes by recent high-throughput sequencing studies [10, 21, 24, 29, 53–62]; however, those studies also did not run kit blanks to assess the possible contamination. More convincing are studies that have cultured these groups from corals [23, 63–72] (e.g., see matching sequences listed in Table 3). The fact that so much of the coral microbiome literature is overshadowed by the uncertainty of whether these associates are real or contaminants makes it imperative to include kit blanks in future sequence surveys, as well as to focus future work on cultivation and confirmative microscopy of these bacterial taxa.

### Core bacterial associates

The *Propionibacterium* OTU was found in 92% of the 51 coral samples and was present across all 7 coral hosts (Table 1). This OTU was included in the 100% conserved core of individual species *Anthothela* sp., *Lo. pertusa*, *P. placomus*, *Pr. resedaformis*, and *Pr. pacifica*. Of the 9 *Propionibacterium* ASVs, the most common was ASV_50 which was 100% identical to the OTU sequence and was found in 63% of the samples (Table 2). The presence of *Propionibacterium* has previously been noted in the conserved cores (90–100% sample inclusion) of a number of stony corals: *Acropora hycinthus*, *Acropora muricata*, *Acropora rosaria*, *Coelastrea aspera*, and *Porites lutea* [16, 54, 56]. Using less stringent requirements to define the conserved core, *Propionibacterium* has also been described as a rare but consistent endosymbiont in stony corals *Acropora granulosa*, *Leptosiris* spp., and *Montipora capitata* and has been detected at low relative abundance in a number of other stony coral species [29]. Similar sequences were found from a number of coral studies, including one cultured isolate (Table 3). The fact that this *Propionibacterium* sequence was found in both stony corals (order *Scleratinia*) and soft corals (order *Alcyonacea*) raised the question of whether this putative symbiont might also be found in other members of class *Anthozoa*–like anemones (order *Actiniaria*) or zoanthids (order *Zoantharia*). *Propionibacterium* was a minor member of the core microbiome of the anemone *Aiptasia* [73] and was present in anemones *Actinia equina*, *Anemonia viridis* [74], and *Edwardsiella andrillae* [75]. *Propionibacterium* sequences were also detected in the microbiomes of zoanthids *Palythoa caribaeorum* [55] and *Palythoa australiae* [76]. Based on the literature, *Propionibacterium* appears to be conserved not just in corals but across class *Anthozoa*. However, given the recent finding that *Propionibacterium* is also a dominant contaminant in extraction kits [52] and most of these studies did not include kit controls, this finding should be confirmed by non-sequence-based methods, including cultivation and microscopy.

A single *Betaproteobacteria* OTU from the family *Comamonadaceae* was conserved in almost 70% of the samples. Greengenes [40, 41] identified the sequence as *Limnohabitans*, a relatively new genus [77], but RDP Classifier [78] identified the OTU as *Acidovorax*. Aligning the OTU sequence against representatives from both genera revealed that for the 328-bp length of the OTU, there were only 3 bp differences between *Limnohabitans* and *Acidovorax*; however, in all cases, OTU 4483490 shared the same base as *Acidovorax*. Further, OTU 4483490 shared 99% identity over 306 bp with an *Acidovorax* clone from coral *Stephanocoenia intersepta* (Table 3; [79]). *Acidovorax* has been detected in association with tropical stony corals, often as the most abundant OTU [60, 79–81] and as a rare associate of a temperate soft coral [21]. Some strains of *Acidovorax* are capable of heterotrophic denitrification of nitrate, so this bacterium may play a role in coral nitrogen cycling [82].

The possibility of complete nitrogen cycling within corals by bacterial associates has been previously hypothesized based on individual coral core microbiome compositions [11, 28]. A similar hypothesis could be sketched based on several of the conserved OTUs identified in this study. The *Bradyrhizobiaceae* OTU had over 100 identical matches to nitrogen-fixing *Bradyrhizobium* strains from root nodules. *Methylobacterium* sequences have been noted as abundant or core members of other tropical stony coral microbiomes [56, 60] and similar to *Bradyrhizobiaceae*; some *Methylobacteriaceae* have been found to form root nodules and act as nitrogen-fixing symbionts [83]. Fixed nitrogen in the form of ammonia could then be converted to nitrate by the *Pirellulaceae* [84]. From there, nitrate could undergo ammonification by the *Campylobacterales* [85] or alternately undergo denitrification by *Kiloniellales* [86] or *Acidovorax* [82]. This combination of conserved bacterial OTUs could indicate the importance of nitrogen cycling to deep-sea corals.

*Gammaproteobacteria* are common members of coral microbiomes [2, 87–89], and all 7 conserved gammaproteobacterial OTUs were similar to the previously identified coral associates (Table 3). *Acinetobacter*, *Pseudomonas*, and *Vibro* have been hypothesized to play a role in oil degradation to benefit the holobiont [10, 90]. Supporting this idea is the finding that flocculent material coating deep-sea soft corals impacted by an oil spill was dominated by *Acinetobacter* and *Pseudomonas* [91]. *Acinetobacter*, *Pseudomonas*, and *Vibrio* have also been implicated in the degradation of dimethylsulfoniopropionate (DMSP)/dimethylsulfide (DMS) [92], suggesting a role in sulfur cycling. Previously, zooxanthellae were credited with the production of DMSP in corals; however, recent research has shown that aposymbiotic corals also are capable of making DMSP, indicating relevance to all corals not just those in the photic zone [93]. *Enterobacteriaceae* are found in healthy tropical corals [24, 94], and their conserved presence in deep-sea corals which are not directly impacted by sewage suggests that they have a role other than as an indicator of poor water quality [95]. The conservation of specific OTUs from gammaproteobacterial families *Enterobacteriaceae*, *Moraxellaceae*, *Pseudomonadaceae*, *Vibrionaceae*, and *Xanthomonadaceae* and their common occurrence in shallow-water corals (Table 3) implies functional importance to the coral holobiont that requires further study.

Three OTUs belong to the order *Phycisphaerales* (phylum *Planctomycetes*) and had 99–100% identity with uncultured bacteria collected from deep ocean waters [96, 97]. The family *Phycisphaeraceae* within this same order was found to be associated with tropical stony corals and more abundant under highly variable environmental conditions [98]. However, no similar sequences to these *Phycisphaerales* OTUs appear to be associated with tropical corals, possibly suggesting a role specific to cold-water corals.

### *Endozoicomonas*

The rare *Endozoicomonaceae* sequences (more properly, *Hahellaceae* [99]) found associated with *Lo. pertusa* and *Anthothela* spp. are very similar to the sequences previously found associated with shallow-water corals and are clearly partitioned by whether the host is a stony coral (order *Scleractinia*) or a soft coral (order *Alcyonacea*) (Fig. 1). This is supported by a recent study on five Mediterranean soft corals that also found *Endozoicomonas*-affiliated sequences formed a phylogenetic relationship that mirrored that of the hosts’ systematic classification [5]. A recent genomic comparison of functional capabilities of seven strains of coral-associated *Endozoicomonas* indicated that stains had differing abilities, and therefore, divergent genotypes are expected to have different specializations [100]. However, the enrichment of particular functional genes across all strains suggested potential roles centering on carbohydrate cycling and amino acid synthesis, with some strains also contributing vitamins, cofactors, and pigments [100].

*Endozoicomonas* have been found to dominate coral microbiomes in shallow waters, regardless of the host being tropical or temperate, stony or soft [18, 21–23, 25, 101]. Is the rarity in deep-sea corals an artifact of methodology or a reflection of environmental restriction? *Acropora millepora* nearer to natural CO_2_ seeps (pH range 7.28–8.01) had 50% less *Endozoicomonas* than corals at a control site (pH range 7.91–8.09) [102]. The *Lo. pertusa* sites had pH values 7.79–7.86 [13], and the deep-sea soft corals came from sites with pH ranges of 7.94–8.15 [103]. Three of the four cultured type strains of *Endozoicomonas* that have been isolated from shallow corals (*E. montiporae*, *E. euniceicola*, and *E. gorgoniicola*) all have an optimal growth pH of 8.0 [48, 104], while the fourth, *E. acroporae*, has an optimum of pH 7.0 [105]. A second environmental factor could be temperature: the cultured type strains have a minimum growth temperature of 15–20 °C and an optimal range of 22–30 °C; the deep-sea coral habitats sampled in this study had temperatures ranging from 5 to 11 °C (Additional file 1). It is possible that separately or in combination, the lower pH and lower temperatures found in deep-sea coral environments limit the growth of coral-associated *Endozoicomonas*.

### Bacterial community composition

The Binary Sorenson Dice index is unweighted and does not take phylogenetic relationships into account, so separation of coral microbiomes into groups based on host genus is driven by the presence/absence of OTUs (Fig. 2). This makes sense since the presence/absence analyses better reflect the importance of rare OTUs, which make up the majority of these coral-associated bacterial communities. The previous finding by Lawler et al. [11] that the two *Anthothela* spp. microbiomes were not significantly different suggested that conservation of bacterial communities at the host genus rather than species level might be a trend for deep-sea corals. However, in spite of the two *Primnoa* species clustering together, there were significant differences between their microbiomes (*P*_PERMANOVA_ = 0.0005, *t* = 1.9316, 5645 unique permutations). When examined using a weighted Unifrac dissimilarity matrix which takes into account both the abundance of taxa and phylogenetic relationships, *Pr. pacifica* and *Pr. resedaeformis* microbiomes separate by species [30]. The two *Anthothela* species had a very low sequence divergence from each other [11], suggesting the similarity in their microbiomes could be due to how recently the host species diverged, in contrast to the two *Primnoa* species that inhabit the Atlantic and Pacific Oceans respectively [106]. Alternately, it may be that limitations of molecular markers for discriminating soft coral taxonomy may be complicating the host resolution in *Anthothela* spp. [107]. Regardless, the host is consistently the dominant driver of deep-sea coral microbiome structure rather than the environment.

## Conclusions

- This meta-analysis of bacterial microbiome datasets from 7 deep-sea corals revealed 23 highly conserved OTUs and an additional 2 unique ASVs of which 80% were highly similar to the sequences previously identified as coral-associated.
- Many of these sequences are conserved across two orders of corals, suggesting conservation across class *Anthozoa* and highlighting the bacterial groups for future targeted study.
- The conserved OTUs included a combination of *Bradyrhizobiaceae*, *Methylobacteriaceae*, *Pirellulaceae*, *Campylobacteriales*, *Kiloniellales*, and *Acidovorax*, which have the functional possibility of a complete nitrogen cycle. This may indicate the importance of bacterial symbionts to nitrogen cycling in deep-sea corals.
- Unlike the other conserved OTUs, *Phycisphaerales* have not been detected in tropical corals, possibly suggesting a role specific to cold-water corals.
- Rare *Endozoicomonaceae*/*Hahellaceae* sequences are clearly segregated by whether the host is a stony coral (order *Scleractinia*) or a soft coral (order *Alcyonacea*), and the finer clustering pattern reflects the hosts’ phylogeny.
- It is possible that the rarity of *Endozoicomonaceae*/*Hahellaceae* sequences in deep-sea corals is due to the lower pH and lower temperatures found in deep-sea coral environments limiting the growth of coral-associated *Endozoicomonas*.
- Deep-sea coral microbiomes are not consistently organized at the level of host genus, but the host is consistently the dominant driver of deep-sea coral microbiome structure rather than the environment.

## Additional Files

Additional file 1: Coral samples and corresponding environmental data. Table listing the 66 coral samples, their full sample ID, collection location, depth, latitude, longitude, water temperature, and salinity. (PDF 106 kb)

Additional file 2: Workflow with commented scripts and parameters. System information, scripts, and comments for each step in the QIIME analysis to produce OTUs and the DADA2 analysis to produce ASVs. (TXT 52 kb)

Additional file 3: Non-rarefied OTU table. List of OTUs organized by sample and coral host. Includes OTU abundance per sample, reference sequence for each OTU, and the taxonomy. (XLSX 985 kb)

Additional file 4: Filtered ASV table. List of ASVs organized by sample and coral host, after the removal of unclassified sequences. Includes ASV abundance per sample, reference sequence for each ASV, and the taxonomy. (XLSX 1270 kb)

Additional file 5: Summary statistics for sequencing 16S rRNA genes from 51 coral samples. Numbers of sequences per sample, and post-rarefaction to 4287; the calculated number of OTUs per sample; and diversity metrics Chao1, Shannon, Simpson Evenness, and Inverse Simpson. (PDF 95 kb)

## Data Availability

The datasets supporting the conclusions of this article are available in the NCBI Sequence Read Archive under BioProjects PRJNA296835, PRJNA297333, PRJNA305617, and PRJNA348705.
