# Supplementary material for: Microbiomes of stony and soft deep-sea corals share rare core bacteria
Source: Microbiome. 2019 Jun 10;7:90. doi: 10.1186/s40168-019-0697-3 (PMC6558771; doi:10.1186/s40168-019-0697-3)
Supplement: Supplementary file 1 — Coral samples and corresponding environmental data. Table listing the 66 coral samples, their full sample ID, collection location, depth, latitude, longitude, water temperature, and salinity. (PDF 106 kb) [file 40168_2019_697_MOESM1_ESM.pdf]

Additional File 1: Coral samples and corresponding environmental data. Highlighted samples were not included in the diversity analyses due to low number of sequencing reads. References for prior analyses of coral groups:

Anthothela grandiflora, *Anthothela* sp., *Anthothela* ND, and *Lateothela grandiflora* [Lawler et al., 2016, doi:10.3389/fmicb.2016.00458]

Lophelia pertusa [Kellogg et al., 2017, doi:10.3389/fmicb.2017.00796]

Paramuricea placomus [Kellogg, et al., 2016, doi:10.7717/peerj.2529]

Primnoa pacifica and *Primnoa resedaeformis* [Goldsmith et al., 2018, doi: 10.1038/s41598-018-30901-z]

Coral	Sample ID	Collection Location	Depth (m)	Latitude	Longitude	Temp (°C)	Salinity
Anthothela grandiflora	ROV-2012-NF-13-Q6	Baltimore Canyon	434	38.161487	-73.856465	7.1	35.1
Anthothela grandiflora	ROV-2012-NF-13-Q7	Baltimore Canyon	432	38.161070	-73.856163	7.5	35.1
Anthothela grandiflora	ROV-2012-NF-15-Q6	Baltimore Canyon	416	38.173512	-73.841965	7.2	35.1
Anthothela grandiflora	ROV-2012-NF-15-Q7	Baltimore Canyon	457	38.175122	-73.845125	6.8	35.1
Anthothela grandiflora	ROV-2012-NF-16-Q7	Baltimore Canyon	436	38.181985	-73.861092	6.3	35.0
Anthothela grandiflora	ROV-2012-NF-17-Q6	Baltimore Canyon	575	38.118832	-73.847417	5.7	35.0
Anthothela grandiflora	ROV-2012-NF-17-Q7	Baltimore Canyon	575	38.118210	-73.847688	5.7	35.0
Anthothela grandiflora	ROV-2012-NF-18-Q7	Baltimore Canyon	679	38.117922	-73.845465	5.1	35.0
Anthothela grandiflora	ROV-2013-RB-686-Q4	Norfolk Canyon	581	37.054690	-74.603935	5.9	35.0
Anthothela grandiflora	ROV-2013-RB-687-Q5	Norfolk Canyon	606	37.054808	-74.578777	5.7	35.0
Anthothela grandiflora	ROV-2013-RB-688-Q1	Norfolk Canyon	559	37.024297	-74.588163	5.8	35.0
Anthothela grandiflora	ROV-2013-RB-688-Q5	Norfolk Canyon	560	37.024247	-74.588199	5.8	35.0
Anthothela sp.	ROV-2012-NF-18-Q6	Baltimore Canyon	524	38.118158	-73.849030	5.5	35.0
Anthothela sp.	ROV-2013-RB-686-Q5	Norfolk Canyon	581	37.054699	-74.603939	5.9	35.0
Anthothela sp.	ROV-2013-RB-687-Q3	Norfolk Canyon	594	37.054881	-74.577786	5.6	35.0
Anthothela sp.	ROV-2013-RB-688-Q2	Norfolk Canyon	474	37.023429	-74.592413	6.5	35.0
Anthothela sp.	ROV-2013-RB-688-Q4	Norfolk Canyon	557	37.024201	-74.588153	5.8	35.0
Anthothela ND	ROV-2012-NF-01-Q7	Baltimore Canyon	451	38.149448	-73.837895	6.4	35.1

Anthothela ND	ROV-2012-NF-02-Q7	Baltimore Canyon	401	38.144950	-73.834483	6.8	35.1
Anthothela ND	ROV-2012-NF-16-Q6	Baltimore Canyon	435	38.181962	-73.860835	5.7	35.0
Anthothela ND	ROV-2013-RB-687-Q4	Norfolk Canyon	704	37.053907	-74.580567	5.3	35.0
Lateothela grandiflora	ROV-2013-RB-686-Q2	Norfolk Canyon	480	37.058587	-74.605852	6.7	35.1
Lateothela grandiflora	ROV-2013-RB-688-Q3	Norfolk Canyon	474	37.023538	-74.592445	6.4	35.0
Lophelia pertusa	ROV02Q1	Gulf of Mexico (VK826)	490	29.170270	-88.013202	8.2	35.0
Lophelia pertusa	ROV02Q2	Gulf of Mexico (VK826)	490	29.169867	-88.013117	8.2	35.0
Lophelia pertusa	ROV03Q3	Gulf of Mexico (VK826)	487	29.169052	-88.013330	8.6	35.0
Lophelia pertusa	ROV05Q2	Gulf of Mexico (VK906)	403	29.073780	-88.379637	9.8	35.2
Lophelia pertusa	ROV06Q3	Gulf of Mexico (VK906)	413	29.071167	-88.375190	11.0	35.3
Lophelia pertusa	3731K3	Gulf of Mexico (VK906)	397	29.068632	-88.377852	10.7	35.3
Lophelia pertusa	ROV07Q1	Gulf of Mexico (WFS1)	504	26.207422	-84.727083	8.2	35.0
Lophelia pertusa	ROV08Q3	Gulf of Mexico (WFS1)	537	26.197992	-84.732340	8.0	34.4
Lophelia pertusa	ROV09Q1	Gulf of Mexico (WFS1)	543	26.204765	-84.731838	8.3	35.0
Lophelia pertusa	3705K3	Atlantic	751	28.775843	-79.616130	7.5	35.0
Lophelia pertusa	3705K6	Atlantic	743	28.775677	-79.616043	7.5	35.0
Lophelia pertusa	3705K10	Atlantic	743	28.775783	-79.615905	7.5	35.0
Paramuricea placomus	ROV-2012-NF-19-Q1	Baltimore Canyon	379	38.151361	-73.838001	6.0	35.0
Paramuricea placomus	ROV-2012-NF-19-Q2	Baltimore Canyon	381	38.151401	-73.837723	5.8	35.0
Paramuricea placomus	ROV-2012-NF-19-Q5	Baltimore Canyon	381	38.151285	-73.837726	5.8	35.0
Paramuricea placomus	ROV-2012-NF-19-Q6	Baltimore Canyon	382	38.152948	-73.837725	6.0	35.0
Paramuricea placomus	ROV-2012-NF-19-Q7	Baltimore Canyon	382	38.151318	-73.837755	5.9	35.0
Primnoa pacifica	AK325	Tracy Arm Fjord, Alaska	12.8	57.888983	-133.31645	4.6	26.9
Primnoa pacifica	AK342	Tracy Arm Fjord, Alaska	12.5	57.888983	-133.31645	4.6	26.9
Primnoa pacifica	AKUT1	Tracy Arm Fjord, Alaska	13.4	57.888983	-133.31645	4.6	26.9
Primnoa pacifica	AKPP1	Tracy Arm Fjord, Alaska	9.8	57.888983	-133.31645	5.0	30.1
Primnoa pacifica	AKPP2	Tracy Arm Fjord, Alaska	13.1	57.888983	-133.31645	5.0	30.1
Primnoa pacifica	AKPP3	Tracy Arm Fjord, Alaska	12.0	57.888983	-133.31645	5.0	30.1
Primnoa pacifica	AKPP4	Tracy Arm Fjord, Alaska	16.2	57.888983	-133.31645	5.0	30.1
Primnoa resedaeformis	ROV-2012-NF-01-Q6	Baltimore Canyon	450	38.149510	-73.837951	6.2	35.1

Primnoa resedaeformis	ROV-2012-NF-02-Q6	Baltimore Canyon	383	38.149088	-73.836225	9.0	35.2
Primnoa resedaeformis	ROV-2012-NF-05-Q6	Baltimore Canyon	443	38.137820	-73.833683	7.4	35.1
Primnoa resedaeformis	ROV-2012-NF-05-Q7	Baltimore Canyon	443	38.137777	-73.833598	7.4	35.1
Primnoa resedaeformis	ROV-2012-NF-06-Q6	Baltimore Canyon	430	38.139116	-73.833355	7.5	35.0
Primnoa resedaeformis	ROV-2012-NF-06-Q7	Baltimore Canyon	431	38.139081	-73.833381	7.5	34.9
Primnoa resedaeformis	ROV-2012-NF-09-Q6	Baltimore Canyon	506	38.151751	-73.839908	7.3	35.1
Primnoa resedaeformis	ROV-2012-NF-09-Q7	Baltimore Canyon	494	38.151416	-73.839820	7.3	35.1
Primnoa resedaeformis	ROV-2012-NF-10-Q6	Baltimore Canyon	500	38.166420	-73.855801	7.6	35.1
Primnoa resedaeformis	ROV-2012-NF-12-Q6	Norfolk Canyon	535	37.068361	-74.648271	6.2	35.0
Primnoa resedaeformis	ROV-2012-NF-12-Q7	Norfolk Canyon	523	37.067373	-74.648941	6.6	35.1
Primnoa resedaeformis	ROV-2012-NF-20-Q1	Norfolk Canyon	434	37.052256	-74.622626	6.3	35.0
Primnoa resedaeformis	ROV-2013-RB-684-Q1	Norfolk Canyon	411	37.068614	-74.644628	10.8	35.5
Primnoa resedaeformis	ROV-2013-RB-684-Q2	Norfolk Canyon	441	37.067677	-74.643226	9.0	35.2
Primnoa resedaeformis	ROV-2013-RB-684-Q3	Norfolk Canyon	441	37.067687	-74.643226	9.0	35.3
Primnoa resedaeformis	ROV-2013-RB-684-Q4	Norfolk Canyon	498	37.071599	-74.649173	6.3	35.1
Primnoa resedaeformis	ROV-2013-RB-684-Q5	Norfolk Canyon	498	37.071597	-74.649191	6.3	35.1
Primnoa resedaeformis	ROV-2013-RB-686-Q3	Norfolk Canyon	479	37.058606	-74.605781	6.6	35.1
Primnoa resedaeformis	ROV-2013-RB-687-Q2	Norfolk Canyon	576	37.054961	-74.578277	5.5	35.0
