# Supplementary material for: Microbiomes of stony and soft deep-sea corals share rare core bacteria
Source: Microbiome. 2019 Jun 10;7:90. doi: 10.1186/s40168-019-0697-3 (PMC6558771; doi:10.1186/s40168-019-0697-3)
Supplement: Supplementary file 5 — Summary statistics for sequencing 16S rRNA genes from 51 coral samples. Numbers of sequences per sample, and post-rarefaction to 4287; the calculated number of OTUs per sample; and diversity metrics Chao1, Shannon, Simpson Evenness, and Inverse Simpson. (PDF 95 kb) [file 40168_2019_697_MOESM5_ESM.pdf]

Additional File 5: Summary statistics for sequencing of 16S rRNA genes from 51 coral samples. *Based on subsampled sequences (n=4,287). NA = not applicable; AVG = average; SD = standard deviation.

Coral	Sample ID	No. of Sequences	No. of OTUs*	Chao 1*	Shannon*	Simpson Evenness*	Inverse Simpson Index*
Anthothela grandiflora	ROV-2012-NF-13-Q6	29,471	26	39	1.50	0.079	2.1
Anthothela grandiflora	ROV-2012-NF-13-Q7	26,050	35	45	2.16	0.103	3.6
Anthothela grandiflora	ROV-2012-NF-15-Q6	19,004	23	32	1.56	0.112	2.6
Anthothela grandiflora	ROV-2012-NF-15-Q7	24,551	38	69	1.86	0.074	2.8
Anthothela grandiflora	ROV-2012-NF-16-Q7	25,055	34	68	1.56	0.062	2.1
Anthothela grandiflora	ROV-2012-NF-17-Q6	26,817	29	59	1.24	0.056	1.6
Anthothela grandiflora	ROV-2012-NF-17-Q7	10,328	39	61	2.47	0.113	4.4
Anthothela grandiflora	ROV-2012-NF-18-Q7	11,964	52	120	2.48	0.080	4.2
Anthothela grandiflora	ROV-2013-RB-686-Q4	24,843	21	32	1.61	0.128	2.7
Anthothela grandiflora	ROV-2013-RB-687-Q5	14,265	44	65	2.52	0.094	4.1
Anthothela grandiflora	ROV-2013-RB-688-Q1	14,274	52	102	1.58	0.033	1.7
Anthothela grandiflora	ROV-2013-RB-688-Q5	298,116	38	55	1.86	0.059	2.2
**AVG**		**43,728**	**36**	**62**	**1.87**	**0.083**	**2.8**
**SD**		**80,374**	**10**	**27**	**0.44**	**0.028**	**1.0**
Anthothela sp.	ROV-2012-NF-18-Q6	13,032	33	59	2.64	0.154	5.1
Anthothela sp.	ROV-2013-RB-686-Q5	32,471	48	74	2.38	0.078	3.7
Anthothela sp.	ROV-2013-RB-687-Q3	4,637	44	67	2.72	0.114	5.0
Anthothela sp.	ROV-2013-RB-688-Q4	254,884	127	340	3.05	0.047	6.0
**AVG**		**76,256**	**63**	**135**	**2.70**	**0.098**	**5.0**
**SD**		**119,655**	**43**	**137**	**0.28**	**0.046**	**0.9**
Anthothela ND	ROV-2012-NF-01-Q7	17,232	31	65	1.26	0.056	1.7
Anthothela ND	ROV-2012-NF-16-Q6	12,426	119	242	0.99	0.010	1.2
Anthothela ND	ROV-2013-RB-687-Q4	10,152	77	167	1.80	0.023	1.8
**AVG**		**13,270**	**76**	**158**	**1.35**	**0.030**	**1.6**
**SD**		**3,615**	**44**	**89**	**0.41**	**0.024**	**0.3**

Lateothela grandiflora	ROV-2013-RB-688-Q3	13,162	357	404	5.48	0.022	8.0
**AVG**		**NA**	**NA**	**NA**	**NA**	**NA**	**NA**
**SD**		**NA**	**NA**	**NA**	**NA**	**NA**	**NA**
Lophelia pertusa	ROV02Q1	47,499	98	160	1.67	0.018	1.8
Lophelia pertusa	ROV02Q2	146,935	50	89	0.85	0.025	1.2
Lophelia pertusa	ROV03Q3	109,986	62	106	1.65	0.027	1.7
Lophelia pertusa	ROV05Q2	65,118	319	481	3.60	0.012	3.8
Lophelia pertusa	ROV06Q3	39,939	37	51	0.89	0.036	1.3
Lophelia pertusa	3731K3	71,226	51	72	1.81	0.045	2.3
Lophelia pertusa	ROV07Q1	122,102	34	64	1.33	0.050	1.7
Lophelia pertusa	ROV08Q3	90,036	39	56	1.38	0.042	1.6
Lophelia pertusa	ROV09Q1	49,968	31	54	1.59	0.073	2.3
Lophelia pertusa	3705K3	47,098	41	56	1.67	0.048	2.0
Lophelia pertusa	3705K6	131,776	41	48	2.08	0.074	3.0
Lophelia pertusa	3705K10	74,767	216	354	4.14	0.030	6.4
**AVG**		**83,038**	**85**	**133**	**1.89**	**0.040**	**2.4**
**SD**		**36,673**	**90**	**139**	**1.00**	**0.020**	**1.4**
Paramuricea placomus	ROV-2012-NF-19-Q1	4,287	106	111	4.00	0.057	6.0
Paramuricea placomus	ROV-2012-NF-19-Q2	7,226	105	122	3.61	0.048	5.1
Paramuricea placomus	ROV-2012-NF-19-Q5	5,589	176	196	4.58	0.029	5.1
**AVG**		**5,701**	**129**	**143**	**4.06**	**0.045**	**5.4**
**SD**		**1,473**	**41**	**46**	**0.49**	**0.014**	**0.5**
Primnoa pacifica	AK325	22,243	150	204	4.16	0.054	8.1
Primnoa pacifica	AK342	35,370	65	118	1.20	0.022	1.4
Primnoa pacifica	AKUT1	23,068	110	192	1.87	0.016	1.8
Primnoa pacifica	AKPP1	43,163	44	103	0.34	0.024	1.1
Primnoa pacifica	AKPP2	44,283	81	158	0.74	0.014	1.2
Primnoa pacifica	AKPP4	22,142	136	193	2.84	0.025	3.4
**AVG**		**31,712**	**98**	**161**	**1.86**	**0.026**	**2.8**

**SD**		**10,569**	**41**	**43**	**1.43**	**0.014**	**2.7**
Primnoa resedaeformis	ROV-2012-NF-01-Q6	22,450	109	174	1.98	0.018	1.9
Primnoa resedaeformis	ROV-2012-NF-02-Q6	17,037	361	444	5.66	0.033	12.0
Primnoa resedaeformis	ROV-2012-NF-05-Q6	23,286	75	117	1.29	0.019	1.4
Primnoa resedaeformis	ROV-2012-NF-05-Q7	11,428	260	357	4.60	0.027	7.1
Primnoa resedaeformis	ROV-2012-NF-06-Q6	7,549	255	302	5.21	0.043	10.9
Primnoa resedaeformis	ROV-2012-NF-06-Q7	14,659	374	447	5.30	0.029	11.0
Primnoa resedaeformis	ROV-2012-NF-09-Q6	6,773	270	298	5.32	0.037	10.1
Primnoa resedaeformis	ROV-2012-NF-09-Q7	5,024	127	143	4.38	0.058	7.3
Primnoa resedaeformis	ROV-2013-RB-684-Q4	4,754	214	309	5.05	0.046	9.8
Primnoa resedaeformis	ROV-2013-RB-684-Q5	5,821	273	312	5.13	0.021	5.8
**AVG**		**11,878**	**232**	**290**	**4.39**	**0.033**	**7.7**
**SD**		**7,116**	**101**	**115**	**1.51**	**0.013**	**3.8**
